# Atypical Presentation of Periprosthetic Joint Infection With Pseudotumor With a Modular-Neck Stem Implant

**DOI:** 10.7759/cureus.28862

**Published:** 2022-09-06

**Authors:** Yonatan Schwartz, Daniel J Sherwood, Eli Kamara

**Affiliations:** 1 Orthopedic Surgery, Montefiore Medical Center, Bronx, USA

**Keywords:** modular-neck stem implant, trunnionosis, adverse local tissue reaction, pseudotumor, periprosthetic joint infection, total hip arthroplasty

## Abstract

A 64-year-old male with a recalled modular-neck stem implant presented with a soft tissue mass in the lateral thigh. Preoperative testing revealed no signs of infection by the 2018 periprosthetic joint infection criteria. MRI revealed a large soft tissue mass around the implant consistent with a pseudotumor, and we performed revision surgery of the femoral component for trunnionosis. One intraoperative culture was positive for infection, and the patient was placed on antibiotics. Six weeks following revision surgery of the femoral component, the patient presented with acute drainage and was diagnosed with an acute on chronic periprosthetic joint infection and underwent explantation of the femoral and acetabular components with the placement of an antibiotic spacer. Cultures revealed identical bacteria from the index procedure. Given the clinical course, this case likely represents adverse local tissue reaction with an atypical presentation of periprosthetic joint infection. This is the first case presentation of an adverse local tissue reaction and superimposed periprosthetic joint infection with normal infection workup, representing an important consideration when differentiating between pseudotumor and periprosthetic joint infection in modular-neck femoral stem implants.

## Introduction

Modular-neck femoral stems were designed to produce increased versatility during surgery allowing for a more accurate reconstruction of hip biomechanics, thereby increasing the function and longevity of the implant in total hip arthroplasty (THA) [1]. In July 2012, ABGII and Rejuvenate modular-neck stems (Stryker, Kalamazoo, Michigan) were recalled due to high revision rates as a result of trunnionosis with excessive metal debris from the modular neck junction [1,2]. At the time of the recall, 30,000 THAs with these stems had already been implanted worldwide [1]. Revision rates are reported as high as 86%, with a majority of the revisions related to extensive corrosion with soft-tissue destruction [2,3].

While much has been reported in the literature on the prevalence of concurrent periprosthetic joint infection (PJI) in metal-on-metal (MoM) implants, few studies explore this topic with regard to trunnionosis. Similar to MoM implants, differentiating between trunnionosis with adverse local tissue reaction (ALTR) and PJI can be extremely challenging [3,4]. Here, we report a patient with a rejuvenate modular neck stem implant who presented with a pseudotumor and normal pre-operative workup by the 2018 PJI criteria [5] and developed an acute on chronic PJI. Given the presenting labs and clinical course, this case likely represents an atypical presentation of a pseudotumor from trunnionosis with concurrent PJI. To our knowledge, this has not been reported and represents an important consideration when differentiating between pseudotumor and PJI in modular-neck femoral stem implants.

## Case presentation

**Figure 1:**
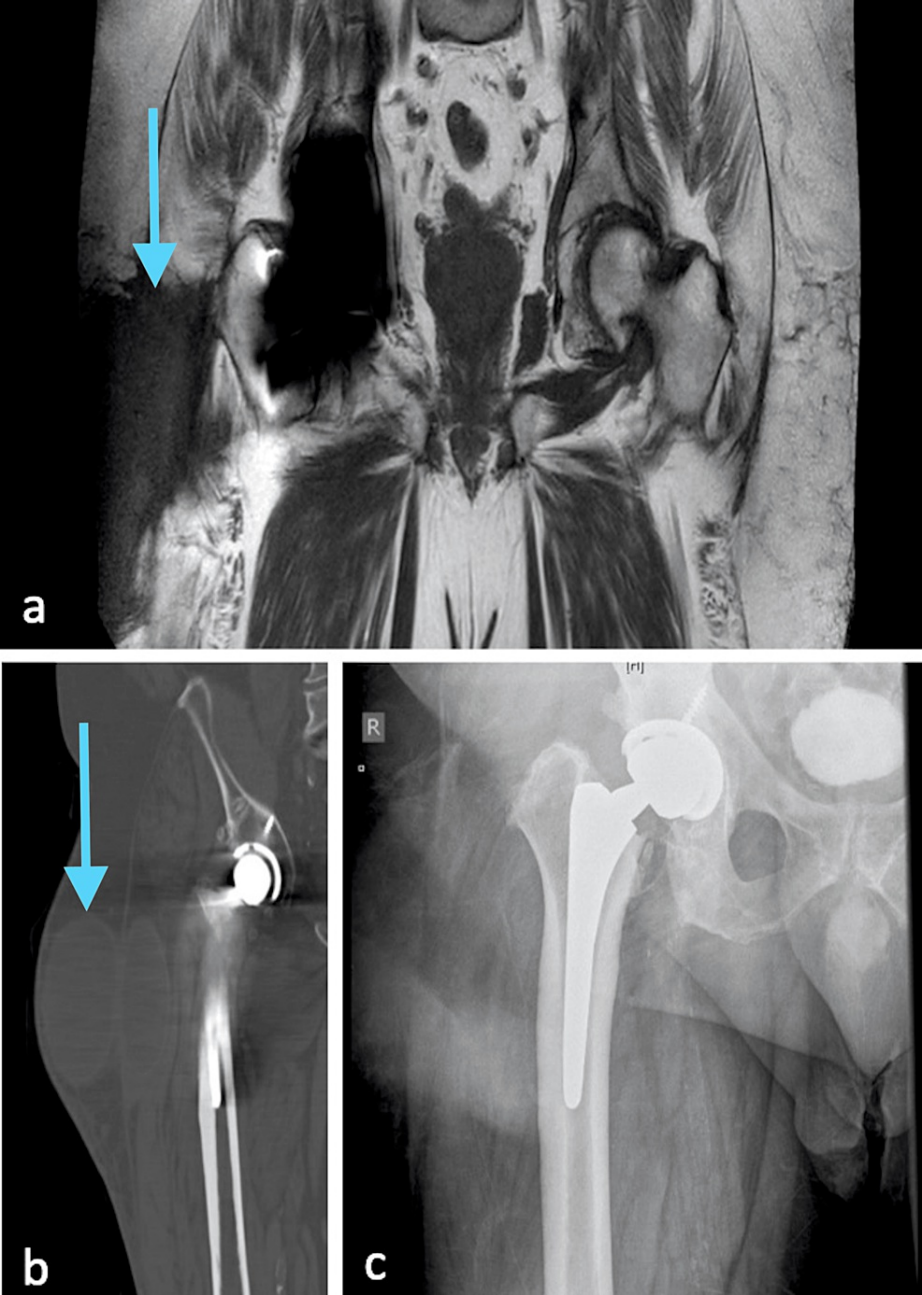
Preoperative imaging MARS-MRI shows a large lobulated cystic mass in the subfascial and extrafascial planes (a). Coronal CT shows the same large lobulated cystic mass (b) and anterior-posterior x-ray (c).

A 64-year-old male with a history of obesity, smoking, chronic obstructive pulmonary disease, hypertension, hyperlipidemia, and pre-diabetes presented to our institution’s emergency department in June 2019. The patient had a right THA eight years prior with a rejuvenate modular-neck stem implant. On presentation, the patient complained of a mass in the right thigh with swelling. On exam, the patient was afebrile, the skin was intact, and there was a palpable mass in the thigh. Labs revealed slightly elevated Chromium levels (1.4mcg/L) with a normal C-reactive protein (CRP) (0.6 mg/dL), erythrocyte sedimentation rate (ESR) (10 mm/hr), D-dimer (0.82 µg/mL), white blood cell (WBC) count (7.6 k/µL) and cobalt (<0.5mcg/L). CT and metal artifact reduction sequences-magnetic resonance imaging (MARS-MRI) revealed a large lobulated cystic mass in the subfascial and extrafascial planes (Figures 1a-1c). Based on the 2018 definition of PJI, the preoperative score was 0 for infection risk [5]. After reviewing the advanced images with interventional radiology, no fluid collection could be identified around the joint capsule, and aspiration was not attempted.

Intraoperatively, a large cavity and soft tissue mass was encountered. The iliotibial band fascia was eroded along with a large section of the vastus lateralis and intermedius. The cavity was excised and sent for pathology. The posterior capsule repair was intact, with no damage to the abductor tendons or muscle. No purulence was encountered in the joint capsule. The acetabular component was noted to be well-fixed and in an appropriate position with no evidence of liner wear. Gross examination of the explanted trunnion revealed blackening at the stem interface, consistent with corrosion. The ceramic head was unable to be disimpacted from the trunnion, therefore this interface was unable to be examined. Given the normal labs (CRP, ESR, D-dimer), lack of systemic signs of infection, and the intraoperative findings consistent with a pseudotumor, the decision was made to proceed with isolated femoral revision. The stem was removed with an extended trochanteric osteotomy. The femoral revision was conducted with placement of a fluted modular stem. Postoperatively, the patient was given Cefazolin until the intraoperative cultures were final.

Pseudotumor formation was confirmed with four intraoperative histopathological specimens; one out of five of the intraoperative cultures was positive for Staphylococcus epidermidis; by the 2018 criteria the patient’s score was now 2 (2-single positive culture), implying the single positive culture was likely a contaminant [5]. Subsequently, the patient was evaluated by Infectious Disease and placed on a course of Bactrim Double Strength (800 mg sulfamethoxazole and 160 mg of trimethoprim) taken twice daily for three weeks.

**Figure 2:**
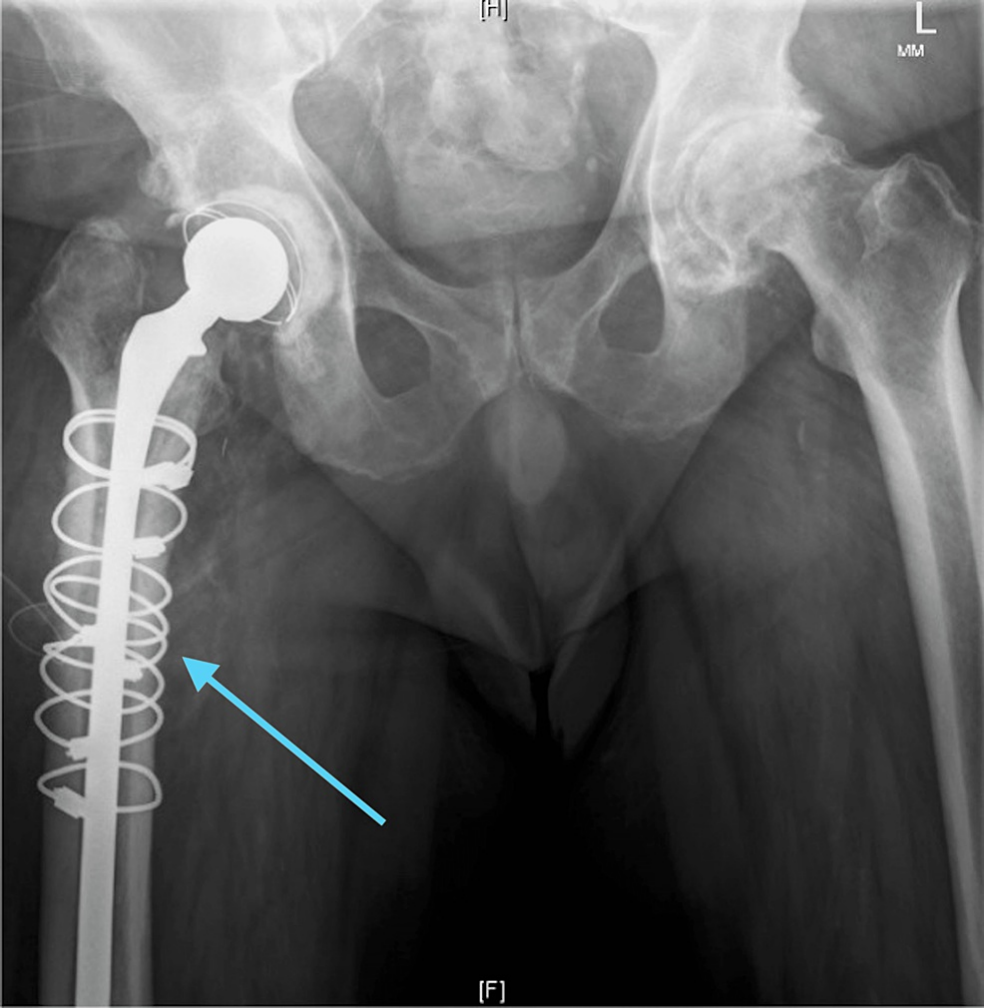
X-ray after explantation of the femoral and acetabular components was performed with placement of an antibiotic spacer.

At the patient’s first clinic visit, approximately two weeks postoperatively, he showed progress and presented with a well-functioning total hip replacement revision. At the patient’s second clinic visit, approximately six weeks postoperatively, the patient had increasing pain, and there was a palpable fluid collection in the right thigh. Due to a concern for an infection given the patient’s positive intraoperative culture, the patient was referred to Interventional Radiology for aspiration. The aspirated fluid was sent for culture and grew S. epidermidis. Given the patient's continued pain, fluid collection, and positive culture, the patient was diagnosed with an acute on chronic PJI and returned to the OR where explantation of the femoral and acetabular components was performed with placement of an antibiotic spacer, as demonstrated by the postoperative x-ray (Figure 2). Intraoperatively, gross purulence was discovered within the hip joint and surrounding soft tissue envelope. One out of four cultures were positive for S. epidermis. Postoperatively, the patient was placed on a course of Cefazolin via peripherally inserted central catheter for six weeks.

Two weeks following completion of the six-week antibiotic course, testing revealed clearance of the infection (CRP 0.7, ESR 10). The patient underwent removal of the antibiotic spacer with replantation THA. He was on oral cefalexin 500mg twice daily for three months post-operatively. At his one-year follow-up, the patient had no evidence of recurrence of infection and had returned to an active lifestyle.

## Discussion

The rejuvenate modular-neck stem implant was recalled in 2012 due to high revision rates [1]. Meftah et al. suggested that the metallurgy mismatch between the cobalt-chromium modular neck in conjunction with the titanium-molybdenum-zirconium-iron stem caused fretting corrosion at the interface leading to a release of metal ions [6]. Walsh et al. discovered a drastic increase in metal ion levels within the hip joint. These ions cause a type II allergic response with the development of an ALTR and pseudotumor [7]. It is important to note that a pseudotumor may develop in the setting of normal Cobalt ion levels and that the final diagnosis must be confirmed with pathology [8,9].

Unfortunately, the diagnosis of infection is particularly challenging with MoM implants as the inflammatory reaction generated from the metal debris can mimic PJI serologically [10,11]. Wyles et al. reported that CRP and ESR are not adequately sensitive or specific for diagnosing PJI in failed MoM hip replacements [12]. MoM implants can also falsely elevate WBC count and neutrophil percentage [11]. While the presence of synovial alpha-defensin has become a strong predictor of PJI, Okroj et al. reported high false-positive results in the setting of ALTR [13]. High suspicion for infection is crucial; Judd et al. reported that 33% (three out of nine) of MoM revision THAs due to pseudotumor were found to be concomitantly infected [14]. Grammatopoulos et al., using only microbiological criteria, found a prevalence of 6.7% for PJI in the failed hip implants in cases not thought to be infected [4]. Synovial fluid WBC count has good sensitivity and specificity but is often falsely positive and its accuracy should be reviewed by a manual count [11]. Leukocyte esterase testing may be appropriate in cases with a high suspicion of infection [15].

It is important to note that no test is 100% accurate in diagnosing PJI [16]. The overlap between ALTR and PJI must be considered carefully when diagnosing a patient. Differentiating between the two, as well as determining whether it is an isolated issue or concomitant, is crucial for treating a patient. This issue is critical for all patients with MoM implants who present with painful hip arthroplasty.

Although post-revision surgeries of MoM implants have increased the likelihood of infection [17], it seems probable the patient had been infected before the first operation, as the bacterial infectant, S. epidermidis, had been positive on intraoperative and postoperative cultures. The preoperative and intraoperative criteria for infection based on the 2018 criteria classified the patient as not infected; future guidelines may need to be modified for patients with pseudotumors to assist providers in making the correct diagnosis in the setting of normal inflammatory markers; routine intraoperative histology may be necessary, irrespective of the pre-operative score [4,11]; in the equivocal setting, treating as PJI might be appropriate. As many modular hip implants exist in the population, the possibility of more cases such as this remains; surgeons treating these patients should have a high suspicion of infection until proven otherwise. More research must be done in order to accurately identify and classify cases where pseudotumor and infection may be superimposed.

## Conclusions

Patients with pseudotumors may have an increased risk for concurrent PJI that may not be identified with traditional criteria for diagnosing periprosthetic joint infections. To our knowledge, this is the first case presentation of superimposed PJI with an ALTR from this particular recalled stem. Our case represents an important consideration when differentiating between pseudotumor and periprosthetic joint infection in modular-neck femoral stem implants. Providers should have a high suspicion of superimposed periprosthetic joint infection in the setting of ALTR.
